# Chromium Picolinate Protects against Testicular Damage in STZ-Induced Diabetic Rats via Anti-Inflammation, Anti-Oxidation, Inhibiting Apoptosis, and Regulating the TGF-β1/Smad Pathway

**DOI:** 10.3390/molecules28227669

**Published:** 2023-11-20

**Authors:** Hongxing Zheng, Yingjun Hu, Mengli Shao, Simin Chen, Shanshan Qi

**Affiliations:** 1 School of Biological Science and Engineering, Shaanxi University of Technology, Hanzhong 723000, China; zhenghongxing100@126.com (H.Z.); huyingjun202103@163.com (Y.H.); shaomengli9722@163.com (M.S.); csm2052617@163.com (S.C.); 2 State Key Laboratory of Qinba Biological Resources and Ecological Environment, Hanzhong 723000, China; 3 Shaanxi Black Organic Food Engineering Technology Research Center, Hanzhong 723000, China; 4 Qinba Mountain Area Collaborative Innovation Center of Bioresources Comprehensive Development, Hanzhong 723000, China; 5 Shaanxi Province Key Laboratory of Bioresources, Hanzhong 723000, China; 6 Shaanxi Daoerfeng Biotechnology Company, Hanzhong 723000, China

**Keywords:** chromium picolinate, diabetes, testicular damage, TGF-β1/Smad pathway

## Abstract

Chromium picolinate (CP) is an organic compound that has long been used to treat diabetes. Our previous studies found CP could relieve diabetic nephropathy. Thus, we speculate that it might have a positive effect on diabetic testicular injury. In this study, a diabetic rat model was established, and then the rats were treated with CP for 8 weeks. We found that the levels of blood glucose, food, and water intake were reduced, and body weight was enhanced in diabetic rats after CP supplementation. Meanwhile, in CP treatment groups, the levels of male hormone and sperm parameters were improved, the pathological structure of the testicular tissue was repaired, and testicular fibrosis was inhibited. In addition, CP reduced the levels of serum inflammatory cytokines, and decreased oxidative stress and apoptosis in the testicular tissue. In conclusion, CP could ameliorate testicular damage in diabetic rats, as well as being a potential testicle-protective nutrient in the future to prevent the testicular damage caused by diabetes.

## 1. Introduction

Diabetes is currently one of the three major non-communicable diseases, and the International Diabetes Federation predicts that the number of patients with diabetes will reach nearly 592 million in 2035 [1]. In addition to high blood glucose, diabetes has many other complications [2], such as diabetic retinopathy, diabetic nephropathy, and diabetic cardiomyopathy. 

Diabetic testicular dysfunction is one of the most important and common complications in male diabetic patients [3]. About 90% of male diabetes patients suffer from varying degrees of reproductive dysfunction and fertility problems, which seriously affect the patients’ physical and mental health and quality of life [4,5]. The characteristics of diabetic testicular dysfunction include changed testicular morphology, decreased spermatogenic cells number, spermatogenic dysfunction, and sex hormone disorder [6,7].

Oxidative stress, inflammation, and apoptosis have been reported to be closely related to the occurrence and development of diabetic testicular dysfunction [8,9,10]. In the process of oxidative stress, the increase in ROS damages testicular cells and leads to testicular spermatogenic dysfunction. Additionally, high levels of ROS can also lead to mitochondrial and DNA damage of sperm, and induce spermatogenic cell apoptosis [11,12].

Testicular interstitial fibrosis also causes testicular dysfunction and is a necessary process in the development of diabetic testicular dysfunction [13]. Testicular interstitial fibrosis can destroy the testicular spermatogenic environment, affect testosterone secretion, spermatogenesis and sperm quality, thus causing testicular dysfunction, male infertility and sexual dysfunction [14]. Transforming growth factor β1 (TGF-β1) is an important factor driving tissue fibrosis and can directly activate Smad signal transduction, thus triggering the over-expression of pro-fibrosis genes; the TGF-β1/Smad pathway is closely related to the diabetes-induced renal fibrosis as well as testicular interstitial fibrosis [15,16].

Studies have found that chromium (III) deficiency is closely related to diabetes [17]. Chromium picolinate (CP), known as chromium picolinate 2-carboxylate, is an organic compound, which has been widely used in hypoglycemic functional foods as well as medicine that treats diabetes [18]. It is used as a raw material to supplement Cr in the field of medicine. It has been reported that chromium picolinate can reduce blood glucose and promote the recovery of morphology, structure and function of damaged islet β cells [19]. Our previous study has found that CP has a protective effect on diabetic nephropathy [20]. Considering the anti-oxidant, anti-inflammatory, and anti-fibrosis properties of CP, we speculated that it might have a protective effect on the testicular damage caused by diabetes.

In the present study, we establish a diabetic rat model and evaluate the protective effect of CP on testicular damage in diabetic rats and its underlying mechanism. This study will provide a basis for using CP in the treatment and nutritional intervention of diabetic testicular damage.

## 2. Results

### 2.1. Effects of Chromium Picolinate on Blood Glucose, Body Weight, Food, and Water Intake of DM Rats

**Figure 1:**
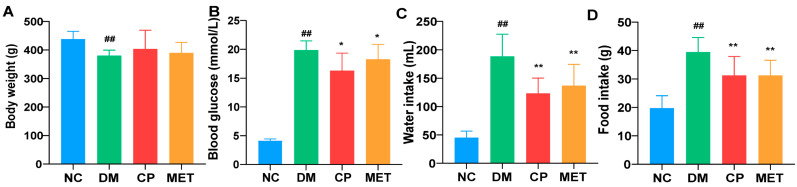
Levels of blood glucose, body weight, food intake, and water intake in each group. NC, normal control group; DM, diabetic model group; CP, DM rats treated with CP (5 mg∙kg^−1^∙d^−1^); and MET, DM rats treated with metformin (200 mg∙kg^−1^∙d^−1^). (**A**) Body weight; (**B**) blood glucose; (**C**) food intake; (**D**) water intake. *n* = 10/group. All data are presented as the Mean ± SD. ANOVA and Tukey’s test were used to analyze the significant differences between any two groups. In comparison to the NC group, (^##^) *p* < 0.01. In comparison to the DM group, (**) *p* < 0.01 and (*) *p* < 0.05.

The influence of chromium picolinate on body weight, water consumption, and food intake of DM rats is shown in Figure 1. Eight weeks of chromium picolinate treatment significantly reduced the blood glucose, the amount of water, and food intake of DM rats (blood glucose, CP vs. DM, *p* < 0.05; food intake and water intake, CP vs. DM, *p* < 0.01). In DM rats, the body weight was lower compared to NC (*p* < 0.01), whereas the body weight of DM rats was not affected by chromium picolinate. No significant body weight difference existed between DM and CP group. There were no significant differences in the levels of blood glucose, body weight, food intake, and water intake between CP and MET groups.

### 2.2. Effects of Chromium Picolinate on the Testicular Index and Sperm Quality of DM Rats

**Figure 2:**
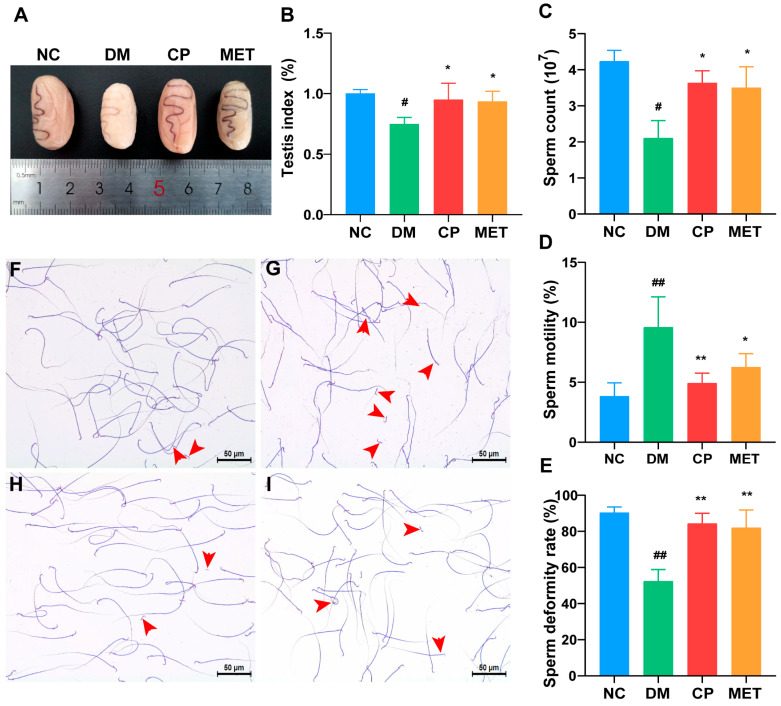
Testicular index, sperm morphology, and sperm quality of rats in each group. (**A**) Testicular appearance; (**B**) testicular index; (**C**) sperm count; (**D**) sperm motility; (**E**) sperm deformity rate; (**F**) sperm morphology in the NC group; (**G**) sperm morphology in the DM group; (**H**) sperm morphology in the CP group; (**I**) sperm morphology in the MET group, magnification of 200×. The red arrows indicate malformed sperm. *n* = 10/group. All data are presented as the Mean ± SD. ANOVA and Tukey’s test were used to analyze the significant differences between any two groups. In comparison to the NC group, (^##^) *p* < 0.01 and (^#^) *p* < 0.05. In comparison to the DM group, (**) *p* < 0.01 and (*) *p <* 0.05.

We observed the changes in the testicular organs and sperm quality of rats in each group, as shown in Figure 2. Compared to the NC group, the testicles in the DM group were significantly atrophied and the testicular index was lower (*p* < 0.05), while the testicles in the CP and MET groups were normal in size and testicular index (*p* < 0.05). Compared to the DM group, there was no significant difference in the testicular index between the CP and MET groups. In the NC group, the number of sperm was higher, sperm motility was higher, and the number of malformed sperm was lower (big heads, tail curls, broken tails, double-headed, and double-tailed). Compared with the NC group, the sperm count and motility of the DM group were significantly decreased (*p* < 0.01), and the rate of sperm malformation was significantly increased (*p* < 0.01). After eight weeks CP or metformin supplementation, the sperm count and sperm motility were significantly increased, sperm abnormality rate was significantly decreased (vs. DM group, *p* < 0.01), and there was no significant difference between the two groups.

### 2.3. Effects of Chromium Picolinate on Serum LH, FSH, and Testosterone Levels in DM Rats

**Figure 3:**
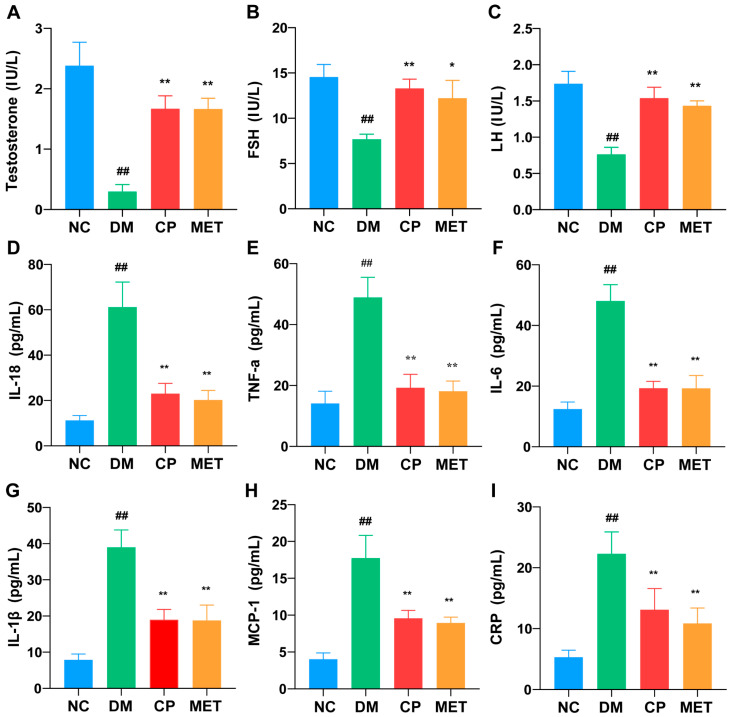
Levels of serum hormones and inflammatory cytokines in each group. (**A**) Level of testosterone in each group; (**B**) level of FSH in each group; (**C**) level of LH in each group; (**D**) IL-18 expression level in each group; (**E**) TNF-α expression level in each group; (**F**) IL-6 expression level in each group; (**G**) IL-1β expression level in each group; (**H**) MCP-1 expression level in each group; (**I**) CRP expression level in each group. *n* = 10/group. All data are presented as the Mean ± SD. ANOVA and Tukey’s test were used to analyze the significant differences between any two groups. In comparison to the NC group, (^##^) *p* < 0.01. In comparison to the DM group, (**) *p* < 0.01 and (*) *p <* 0.05.

As shown in Figure 3A–C, compared to the NC group, testosterone, LH, and FSH levels in the serum were significantly reduced in the DM group (*p* < 0.01), which were enhanced after CP or MET administration (vs. DM group, *p* < 0.01). There was no significant difference between the two groups. Thus, chromium picolinate could increase LH, FSH, and testosterone levels in diabetic rats.

### 2.4. Effects of Chromium Picolinate on Serum Inflammatory Cytokine Levels in DM Rats

As illustrated in Figure 3D–I, the DM group showed significantly (*p* < 0.05) higher IL-18, TNF-α, IL-6, IL-1β, MCP-1, and CRP inflammatory cytokines expression levels in the serum compared to the NC group. The serum inflammatory cytokines were decreased in the CP and metformin groups (vs. DM, *p* < 0.01), but there was no significant difference between the two groups. These results reflect that CP could reduce serum inflammatory cytokines in DM rats. 

### 2.5. Effects of Chromium Picolinate on Testicular GSH, SOD, MDA, and CAT Index of DM Rats

**Figure 4:**
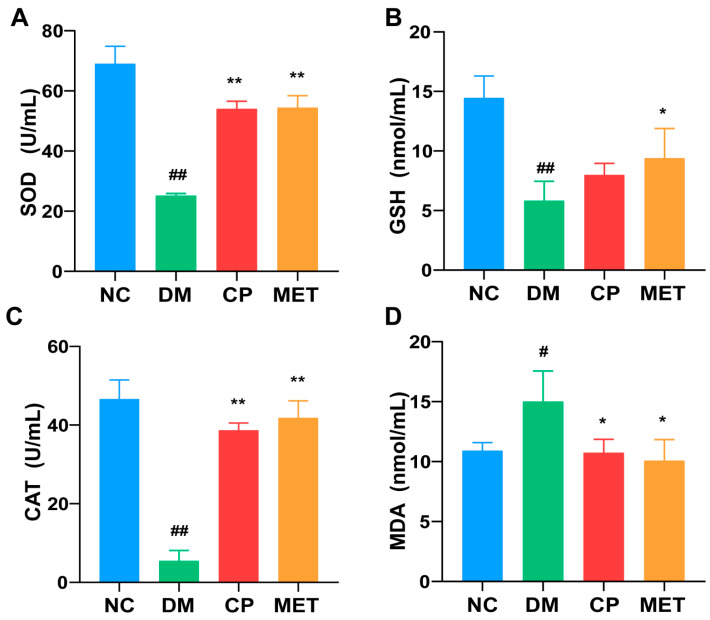
Levels of serum SOD, GSH, CAT, and MDA index in each group. (**A**) SOD level in each experimental group; (**B**) GSH level in each experimental group; (**C**) CAT level in each experimental group; (**D**) MDA level in each experimental group. *n* = 10/group. All data are presented as the Mean ± SD. ANOVA and Tukey’s test were used to analyze the significant differences between any two groups. In comparison to the NC group, (^##^) *p* < 0.01 and (^#^) *p* < 0.05. In comparison to the DM group, (**) *p* < 0.01 and (*) *p <* 0.05.

The data in Figure 4 show a dramatically decrease in the SOD, GSH, and CAT levels in the testis of DM rats. Meanwhile, the MDA level was increased significantly in the DM group (vs. NC, *p* < 0.01). The testicular tissue of the CP-treated groups and metformin group displayed significantly higher SOD and CAT levels (vs. DM, *p* < 0.01), as well as a lower MDA level (vs. DM, *p* < 0.05). There was no significant difference in the testicular GSH, SOD, MDA, and CAT levels between CP group and MET group. This indicates that CP could relieve the testicular oxidative stress of diabetic rats.

### 2.6. Effect of Chromium Picolinate on the Structure of the Testicular Tissue of Diabetic Rats

**Figure 5:**
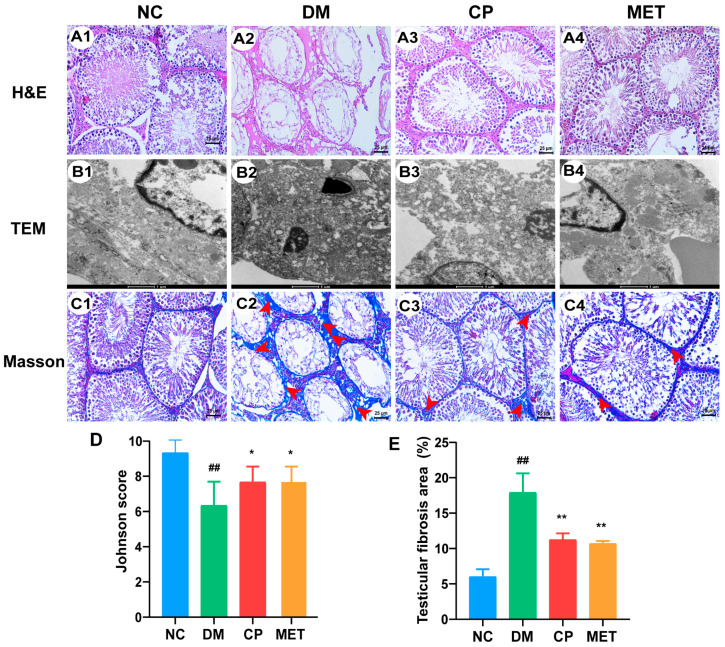
Pathological observation of the testicular tissue in each group. (**A1**–**A4**) HE staining of the testicular tissue in each group, magnification of 200×. (**B1**–**B4**) Testicular ultrastructure observed in each group, magnification of 16,500×. (**C1**–**C4**) Masson’s trichrome staining of the testicular tissue in each group, magnification of 200×. The red arrow shows fibrotic tissue. (**D**) Johnson scores of the testicular tissue’s pathology injury in each group. (**E**) Testicular interstitial fibrosis area (%) in each group. *n* = 10/group. All data are presented as the Mean ± SD. ANOVA and Tukey’s test were used to analyze the significant differences between any two groups. In comparison to the NC group, (^##^) *p* < 0.01. In comparison to the DM group, (**) *p* < 0.01 and (*) *p <* 0.05.

HE staining is shown in Figure 5(A1–A4). In the NC group, no abnormalities were observed in the testicular tissue. The spermatogenic tubules were arranged neatly, with a large number of spermatocytes, spermatogonium, and sperm cells clearly visible. Contrarily, in the DM group, the spermatogenic tubules were irregular, interstitial cells were reduced, and spermatogenic cells were separated from the basement membrane. In addition, there were only a few spermatogonium and spermatocytes distributed in the seminiferous tubules, and the arrangement of the spermatogenic cells were disordered. There were no sperm cells in the seminiferous tubules in the DM group. The mean Johnson score was reduced in the DM group compared to the NC group (*p* < 0.01, Figure 5D). After CP or metformin supplementation, the structure of the testicular tissue was repaired, and the spermatocytes and spermatogonium count also increased in the seminiferous tubules. As indicated in Figure 5D, the Johnson score was up-regulated in the CP- or metformin-treated group (vs. DM, *p* < 0.05), but there was no significant difference in the Johnson score between the CP group and MET group. All data show that the CP repaired the pathological structure of the testicular tissue in diabetic rats.

### 2.7. Effect of Chromium Picolinate on the Ultrastructure of the Testis of DM Rats

In Figure 5(B1), a normal ultrastructure of the testis was observed in the NC group. The cell structure in the seminiferous tubules of the testis was complete, the nuclear membrane was clear and complete, the Sertoli cell nucleus was large, the nucleolus was clear, and the cytoplasm was rich in long rod-shaped mitochondria. In the DM group, the organelles in cytoplasm were dissolved, the nuclear membrane was blurred, the number of organelles decreased significantly, and the mitochondrial crest was broken and reduced (Figure 5(B2)). After CP or metformin treatment, the ultrastructure of the testis turned to be normal, as shown in Figure 5(B3–B4).

### 2.8. Effect of Chromium Picolinate on the Testicular Interstitial Fibrosis of Diabetic Rats

**Figure 6:**
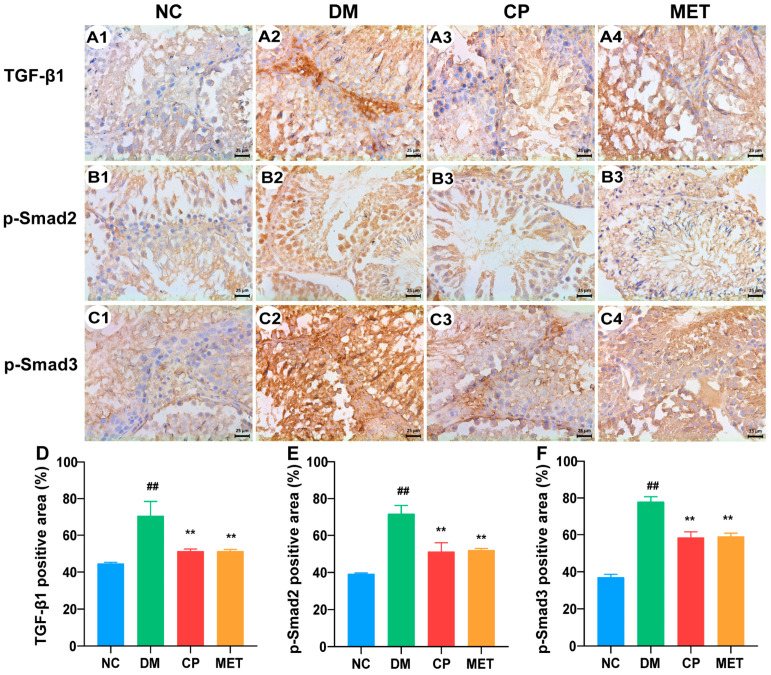
TGF-β1, p-Smad2, and p-Smad3 protein expression in the testicle tissues of each group, with immunohistochemical staining and magnification of 400×. (**A1**–**A4**) TGF-β1 in the testicles of rats in each group. (**B1**–**B4**) p-Smad2 in the testicles of rats in each group. (**C1**–**C4**) p-Smad3 in the testicles of rats in each group. (**D**) TGF-β1-positive staining area in each group. (**E**) p-Smad2-positive staining area in each group. (**F**) p-Smad3-positive staining area in each group. *n* = 10/group. All data are presented as the Mean ± SD. ANOVA and Tukey’s test were used to analyze the significant differences between any two groups. In comparison to the NC group, (^##^) *p* < 0.01. In comparison to the DM group, (**) *p* < 0.01.

Masson’s staining results are shown in Figure 5(C1–C4,E). In the NC group, there was less testicular interstitial fibrosis and a lower percentage of Masson’s-stained positive areas. A large area of testicular interstitial fibrosis was observed in the DM group and a higher percentage of Masson’s-stained positive areas (vs. NC, *p* < 0.01). After CP or metformin administration, the degree of fibrosis in the testicles was significantly decreased, and the percentage of Masson’s-stained positive areas were reduced significantly (CP or MET vs. DM, *p* < 0.01), but there was no significant difference between the two groups. In addition, to investigate the mechanism of CP inhibition of testicular interstitial fibrosis in diabetic rats, immunohistochemical staining for TGF-β1, p-Smad2, and p-Smad3 was conducted (Figure 6). The protein expression levels of TGF-β1, p-Smad2, and p-Smad3 were significantly enhanced in DM rats (vs. NC, *p* < 0.01); they were decreased by eight weeks of CP or metformin treatment. However, there was no significant difference in the protein expression levels of TGF-β1, p-Smad2, and p-Smad3 between the CP group and MET group. These data reveal that CP administration could inhibit the testicular interstitial fibrosis of diabetic rats by down-regulating the testicular TGF-β1/Smads pathway.

### 2.9. Effect of Chromium Picolinate on Bax, Capase-3, Bcl-2, and NF-κB Protein Expression Levels in the Testis of DM Rats

**Figure 7:**
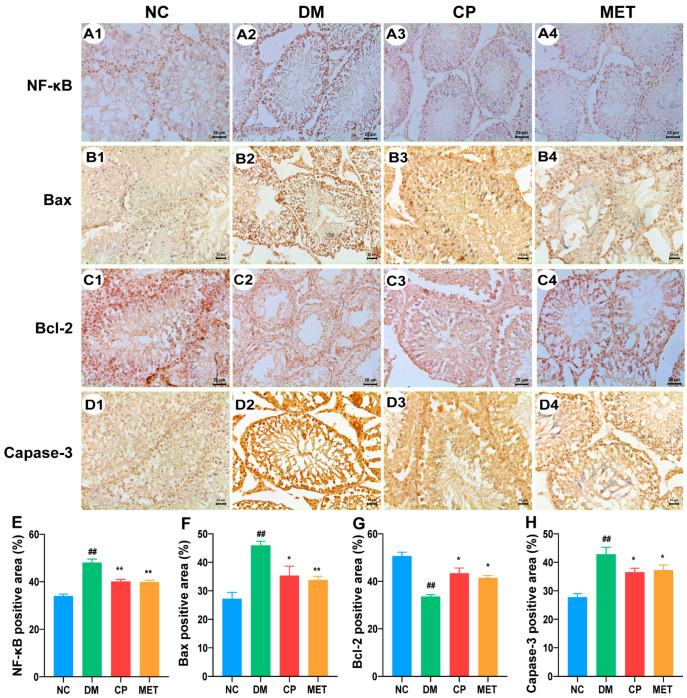
NF-κB, Bax, Bcl-2, and Caspase-3 protein expression in the testicle tissues of each group, with immunohistochemical staining and magnification of 400×. (**A1**–**A4**) NF-κB in the testicles of rats in each group. (**B1**–**B4**) Bax in the testicles of rats in each group. (**C1**–**C4**) Bcl-2 in the testicles of rats in each group. (**D1**–**D4**) Caspase-3 in the testicles of rats in each group. (**E**) NF-κB-positive staining area in each group. (**F**) Bax-positive staining area in each group. (**G**) Bcl-2-positive staining area in each group. (**H**) Caspase-3-positive staining area in each group. *n* = 10/group. All data are presented as the Mean ± SD. ANOVA and Tukey’s test were used to analyze the significant differences between any two groups. In comparison to the NC group, (^##^) *p* < 0.01. In comparison to the DM group, (**) *p* < 0.01 and (*) *p <* 0.05.

As illustrated in Figure 7, compared with NC rats, the protein expression levels of NF-κB, Bax, and Caspase-3 in DM rats’ testis were up-regulated (*p* < 0.01). They were down-regulated by CP or metformin administration (CP or Met vs. DM, *p* < 0.01). In the DM group, the Bcl-2 expression level was reduced (vs. NC, *p* < 0.01), but was increased by CP or metformin treatment (CP or Met vs. DM, *p* < 0.01). However, there was no significant difference in the protein expression levels of NF-κB, Bax, Bcl-2, and Caspase-3 between the CP group and MET group. The above result indicates that CP could regulate apoptosis-related proteins in diabetic rat testis.

## 3. Discussion

Diabetes is a chronic metabolic disease characterized by hyperglycemia [21]. In diabetes, multiple organs and systems are unnormal, accompanied by a variety of complications [22], of which diabetic testicular injury is one of the most common. Diabetic testicular injury is often accompanied by changes in the testicular tissue structure, disorders of male hormone levels, decreased sperm motility, and increased sperm malformation [23]. In this study, an STZ injection was used to a establish diabetic animal models in order to assess the testicle-protective effect and mechanism of chromium picolinate on diabetic rats by chromium picolinate administration.

Since STZ could damage pancreatic islet β cells in a variety of animals and its toxicity is lower than that of alloxan; therefore, it is often chosen to establish diabetic rat models [24]. Yuning Liu et al. selected a 50 mg/kg STZ injection to establish a diabetes model. In the DM group, mice showed specifically polydipsia, polyuria, polyphagia, and weight loss [25]. What is more, the sperm vitality and sperm numbers of the diabetic mice were decreased significantly. In the study of Weiguo He et al., a diabetic rat model was established by using the high-fat diet and streptozotocin injection method. Testosterone, LH, and FSH levels in the serum were significantly decreased in the T2DM group [26]. In addition, the testicular morphology of diabetic rats showed obvious atrophy of the seminiferous tubules, thickened basal membrane of the spermatogenic tubules, and the number of apoptotic cells were increased. The above results are similar to ours. This indicates that STZ not only damages islets to induce diabetes, but also causes testicular damage in diabetic rats. Metformin is a classic drug for the clinical treatment of diabetes. Many studies of diabetic complications choose metformin treatment as the positive control group [27], and studies had revealed metformin has a beneficial effect on diabetes-induced testicular damage [28]. Yuning Liu et al. found that MET (250 mg·kg^−1^·d^−1^) ameliorates testicular injury in male mice with streptozotocin-induced diabetes by regulating the PK2/PKR pathway [25]. Thus, metformin was used as a positive treatment control in this study.

Oxidative stress is an important cause of testicular damage in diabetes, which causes testicular atrophy, sperm defects, germ cell death, and altered levels of reproductive hormones [29]. In high blood glucose, it leads to an excessive accumulation of free radicals, which will attack unsaturated fatty acids leading to lipid peroxidation and then the generation of MDA [30]. MDA plays an important role in oxidative stress and apoptosis, and GSH, SOD, and CAT are crucial antioxidant enzymes [31]. Guang-Jiang Shi et al. found that the levels of SOD, GSH-PX, and CAT in diabetic mice serum of were significantly decreased, while the MDA level was significantly increased with *Lycium barbarum* polysaccharide treatment [32]. These results are consistent with those of this study. The results show that CP could eliminate oxygen free radicals, inhibit lipid peroxidation, and improve the degree of oxidative stress in diabetic rats.

The main function of testicular interstitial cell is to produce androgen. Testosterone, an important male hormone, is essential for spermatogenesis and the improvement of secondary sex characteristics [33]. LH regulates Leydig cell function, and FSH regulates spermatogenesis [34]. When testosterone levels drop in diabetic rats, the interstitial testes and spermatogenic tubules are damaged [35]. In the study of Wafa A. Al-Megrin et al., an Arabica green coffee extract significantly improved testosterone, FSH, and LH levels in high-fat diet/streptozotocin-induced diabetic rats [36]. This is similar to the results of this study. Here, CP significantly increased the levels of testosterone, FSH, and LH in diabetic rats.

Inflammation is widely regarded as the pathogenesis of hyperglycemia and its complications [29,37]. In diabetes, reducing the expression of inflammatory factors can effectively alleviate testicular injury [38]. The over-expression of IL-6 triggers an inflammatory response that suppresses testosterone production and contributes to testicular damage in diabetes patients [39,40]. IL-6, IL-1β, and TNF-α, acting as inhibitors of Leydig cell function, inhibit LH-induced testosterone production [41,42]. Thus, the over-expression of these inflammatory cytokines may lead to testicular injury by affecting Leydig cells, sustentacular cells, and macrophages, and by inhibiting the expression of spermatogenic enzymes (acid phosphatase, lactate dehydrogenase, and gamma-GT). Yin Cheng et al. showed that lycopene could reduce the levels of IL-1α, IL-1β, TNF-α, IL-6, and MCP-1 in rats with lipopolysaccharide-induced testicular injury. This is similar to the results of this study [43]. These results indicate that CP had a beneficial effect on testicle inflammation in diabetic rats by reducing the release of pro-inflammatory cytokines.

Apoptosis is an important mechanism of testicular injury in diabetic animals [44]. Therefore, the inhibition of diabetes-induced apoptosis is considered as a potential treatment for testicular injury. Diabetes increases apoptotic cell death in testicular tissues by up-regulating or down-regulating Bcl-2 family of proteins [45]. It has been shown that hyperglycemia induces apoptosis by disrupting the balance between Bax and Bcl-2 proteins [46]. This imbalance may lead to the release of cytochrome C from the mitochondrial matrix into the cytoplasm, resulting in an increased expression of caspase-3, which promotes DNA enzyme degradation [47]. In addition, Wafa A. al-Megrin et al. showed that, after treatment with green coffee, the content of Bcl-2 in the testis of diabetic rats was increased, while the Bax and Caspase-3 contents were decreased. These results are consistent with those of this study [36]. Therefore, CP may inhibit the apoptosis of testis spermatogenic cells by regulating the expression of apoptosis-related proteins, playing a protective role in testis.

The interstitial fibrosis of testis is a necessary process of diabetic testicular dysfunction, and the fibrosis process is irreversible [14]. The interstitial fibrosis of testis destroys the spermatogenic environment of testis and damages testosterone secretion and spermatogenesis, leading to male sterility and sexual dysfunction [48]. Therefore, preventing testicular fibrosis has become an important way to cure testicular damage in diabetes. TGF-β1 is a ubiquitous cytokine that regulates cell growth [49]. In the testis, it is secreted mainly by the Leydig cells and sustentacular cells of the testis, and acts upon binding to its corresponding receptors by means of both secretion and paracrine [50]. Evidence suggests that transforming growth factor-β-1 is associated with reproductive dysfunction through the activation of testicular fibroblasts and induction of sperm apoptosis [50]. The absence or over-expression of this factor can affect male reproductive function. The activation of TGF-β1 signaling depends primarily on the phosphorylation of Smad protein [51]. When chronic hyperglycemia occurs, Leydig cells and sustentacular cells secrete and synthesize excessive TGF-β1 [13]. TGF-BRII receptor on Leydig cell membranes binds to TGF-β1, and TGF-β1 also binds to TGF-BRI receptor to form trimer complex, which phosphorylates Smad2 and Smad3 [52]. Smad4 combines with phosphorylated Smad2 and Smad3 to form a trimer that enters Leydig cells, regulates the transcription of related genes, and regulates interstitial fibrosis of testis in animals [53]. Masson’s staining results and immunohistochemical staining results show that CP could inhibit inflammation and fibrosis, providing evidence with respect to the potential therapeutic impact on fibrosis-related diseases.

## 4. Materials and Methods

### 4.1. Materials

Chromium picolinate was purchased from TargetMol Chemicals Inc. (Boston, MA, USA); STZ was purchased from Shanghai Aladdin Bio-Chem Technology Co., Ltd. (Shanghai, China).

### 4.2. Animals and Experimental Design

Eight-week-old male SD rats (specifically pathogen-free grade) were purchased from Cheng Du Dashuo Experimental Animal Center (animal production license number: SCXK2020-030). The rats were kept in standardized animal houses laboratory at a controlled temperature of 22 °C ± 2 °C and air humidity of 50% ± 5%. They were fed with standard rat food (AIN93) and drinking purified water freely. The animal study in this research was conducted in the Shaanxi Daoerfeng Biotechnology Institute, and all experimental animal protocols were approved by the Animal Ethics Committee of the Shaanxi Daoerfeng Biotechnology Institute (No. 20211102).

After 7 days of adaptation, the rats were randomly divided into a normal control group (NC), diabetic model group (DM), metformin group (MET, as positive control group), and chromium picolinate group (CP), with 10 rats in each group. Fasting for 12 h, the NC group was administered citrate buffer; the DM group, MET group, and CP group were administered a one-time fasting intraperitoneal injection of 45 mg·kg^−1^ STZ. After 72 h, blood samples were collected from tail vein of rats to detect random blood glucose. When the concentration was higher than 11.1 mmol·L^−1^, the rats were considered as diabetic rats. The normal group and the model group were provided distilled water every day, the metformin group was administered 200 mg·kg^−1^ metformin every day, and the CP group was administered 5 mg·kg^−1^ chromium picolinate every day for eight weeks. Blood glucose and body weight of all rats were measured weekly and recorded. After 8 weeks of treatment, rats were isoflurane anesthesia, testis was taken out and accurately weighed. A blood sample was collected, and the serum was centrifuged (3000 r/min, 10 min) and then stored at −80 °C refrigerator for later.

$$\mathrm{Testis}\;\mathrm{index}=\frac{\mathrm{testiculars}\;\mathrm{weight}}{\mathrm{body}\;\mathrm{weight}\;\mathrm{of}\;\mathrm{rat}}\times 100\%$$

### 4.3. Sperm Parameters: Sperm Motility Sperm Count and Sperm Deformity Rate Assessment

The left epididymis was taken out and put into 100 mL preheated saline at 37 °C. A total of 50 μL semen was taken from the fresh epididymis of rats and incubated in a 0.2 mL centrifuge tube at 37 °C for 30 min waiting for sperm liquefaction. Sperm motility was detected by a computer-assisted semen analysis system (CASA, Song Jing Tianlun Biotechnology Co., Ltd., Nanning, China). The CASA analyzer was operated according to the user guide.

A total of 1–2 drops of fresh sperm suspension were obtained from the epididymis of each rat. After natural drying, methanol was dropped on the slide and fixed for 5 min, dyed with eosin for 30 min, rinsed with running water, and dried at room temperature. Sperm stain slides from each rat were examined at 30 fields of view. The abnormal sperm (head, mid-piece, and tail abnormalities) were viewed by microscope (Leica DM 3000), and total number of sperm and the number of abnormal sperm were counted.

$$\mathrm{Sperm}\;\mathrm{deformity}\;\mathrm{rate}=\frac{\mathrm{the}\;\mathrm{number}\;\mathrm{of}\;\mathrm{abnormal}\;\mathrm{sperm}}{\mathrm{total}\;\mathrm{number}\;\mathrm{of}\;\mathrm{sperm}}\times 100\%$$

### 4.4. Serum LH, FSH, and Testosterone Detection

Serum luteinizing hormone (LH), follicle stimulating hormone (FSH), and testosterone levels were detected based on the operation procedures listed in the ELISA kit (Wuhan Saipei Biotech Co., Ltd., Wuhan, China), by using a microplate reader. 

### 4.5. Inflammatory Cytokines Detection

IL-18, TNF-α, IL-6, IL-1β, MCP-1, and CRP levels in the serum and testis homogenate were detected based on the operation procedures listed in the ELISA kit (Wuhan Saipei Biotech Co., Ltd., Wuhan, China), by using a microplate reader. 

### 4.6. GSH, SOD, MDA, and CAT Index Detection 

Levels of GSH, SOD, MDA, and CAT in the testis homogenate were detected according to the operation procedures of the kits (Wuhan Saipei Biotech Co., Ltd., Wuhan, China) by using a spectrophotometer (YuanXiV-5800, Shanghai, China).

### 4.7. Histological Evaluation of the Testes

The left testis of rats was taken from each rat, and were soaked in 4% paraformaldehyde; 48 h later, testis tissues were dehydrated by gradient ethanol (75%, 80%, 90%, 95%, and 100%) for 1 h each, and soaked in xylene for 15 min. Then, testicular tissues were immersed in paraffin (58 °C) for 3 h. After that, the testicular tissues were embedded in paraffin, and cut into 4 μm thick by Leica RM2235 tissue slicer. The testicular tissue slide was stained with hematoxylin and eosin, and the slides were observed under a microscope. Each testicular slide was observed at randomly selected ten visual fields and the number of spermatogenic cells was counted; and the testicular pathological scores were made by Johnson’s mean testicular biopsy score [29]. Each seminiferous tubule was scored from 0 to 10 points depending on the maturity of the seminiferous tubule epithelium, as listed in Table 1.

### 4.8. Transmission Electron Microscopic Observation of the Testis Ultrastructure

Fresh testicular tissues of rats in each group were fixed in 3% glutaraldehyde solution at 4 °C for 30 min. After the testicular tissue became hard, the tissue mass (1 cm × 1 cm × 1 cm) was repaired with a scalpel, and then rinsed with PBS buffer for 3 times. Then, the tissues were immersed in 1% osmium acid at 4 °C for 1.5 h, and washed with sodium dimetharsenate buffer (0.1 M) for 15 min. The testicular tissues were dehydrated by gradient ethanol successively (30%, 50%, 70%, 80%, 90%, and 100%) for 15 min each time, and then embedded with epoxy resin to prepare ultra-thin sections (70 nm thick). The samples were stained with 0.25% citric lead acid and 0.5% uranium-dioxide acetate. The ultrastructural changes in the testis were observed by transmission electron microscope.

### 4.9. Testicular Interstitial Fibrosis Evaluation

The testicles of each rat were sliced into 5 μm slices and were stained by Masson’s Trichrome staining method. The fibrous connective tissues in the testicular interstices were stained blue after Masson’s staining. Each testicular slide was observed and quantified for ten randomly selected visual fields, and Image Pro Plus 6.0 software was used to measure the fibrotic area and the total area; then, the ratio of the two areas was calculated.

### 4.10. Immunohistochemical Analysis of Apoptosis and Fibrosis-Related Proteins in Testis

Sections of each rat were selected for immunohistochemical staining. A total of 5 μm thick testicular paraffin slides were soaked in xylene, anhydrous ethanol, and 0.3% hydrogen peroxide solution for 15 min. Then, they were placed in a container containing PBS and put in a microwave, treated with microwave for 15 min; then, goat serum was added to the slices and incubated for 30 min at 36 °C. A primary antibody (Bax, Bcl-2, Caspase-3, TGF-β1, Smad 2, and Smad 3) was dropwise added to the slide, and incubated at 36 °C for 1.5 h; after PBS washing, the secondary antibody was dripped on the slides, and incubated at 36 °C for 2 h. Then, the sections were stained with DAB kit (Gefan Biotechnology Company, Shanghai, China), and then sealed with neutral gum. Leica microscopes were used for observation and photography. Ten fields were randomly selected for immunohistochemical staining analysis in each testicular slide and the positive staining areas of Bax, Bcl-2, Caspase-3, TGF-β1, Smad 2, and Smad 3 were analyzed by using Image Pro-Plus 6.0 software.

### 4.11. Data Analysis

The result data were determined using SPSS (version 26.0) statistical analysis. One-way analysis of variance (ANOVA) and Tukey’s test were used to analyze the significant differences between any two groups.

## 5. Conclusions

In conclusion, this study found that CP could ameliorate testicular damage in diabetic rats. It could improve the sperm’s quality, inhibit testicular interstitial fibrosis, repair the morphology structure of testicular tissue, and regulate reproductive hormone levels. The mechanisms are related to anti-oxidation, anti-inflammatory, regulating testicular cell apoptosis-related proteins, as well as inhibiting the TGF-β1/Smad pathway. Thus, the microenvironment of testis can be improved and spermatogenesis be promoted. This could be a potential testicle-protective nutrient in the future to prevent the testicular damage caused by diabetes.

## Figures and Tables

**Table 1:** Johnson’s pathological score of testicular tissues.

Score	Description
1	No cells
2	Sertoli cells without germ cells
3	Only spermatogonia
4	Only a few spermatocytes
5	Many spermatocytes
6	Only a few early spermatids
7	Many early spermatids without differentiation
8	Few late spermatids
9	Many late spermatids
10	Full spermatogenesis

## Data Availability

Data are contained within the article.

## References

[1] Kharroubi A.T., Darwish H.M. (2015). Diabetes mellitus: The epidemic of the century. World J. Diabetes.

[2] King G.L. (2008). The role of inflammatory cytokines in diabetes and its complications. J. Periodontol..

[3] Kyathanahalli C., Bangalore S., Hanumanthappa K., Muralidhara (2014). Experimental diabetes-induced testicular damage in prepubertal rats. J. Diabetes.

[4] He Z., Yin G., Li Q.Q., Zeng Q., Duan J. (2021). Diabetes mellitus causes male reproductive dysfunction: A review of the evidence and mechanisms. In Vivo.

[5] Pavlinkova G., Margaryan H., Zatecka E., Valaskova E., Elzeinova F., Kubatova A., Bohuslavova R., Peknicova J. (2017). Transgenerational inheritance of susceptibility to diabetes-induced male subfertility. Sci. Rep..

[6] Apichakan S., Supatcharee A., Jaturon B., Wannisa S., Sitthichai I. (2018). Testicular histopathology and phosphorylated protein changes in mice with diabetes induced by multiple-low doses of streptozotocin: An experimental study. Int. J. Reprod. Biomed..

[7] Korejo N.A., Wei Q.W., Shah A.H., Shi F.X. (2016). Effects of concomitant diabetes mellitus and hyperthyroidism on testicular and epididymal histoarchitecture and steroidogenesis in male animals. J. Zhejiang Univ. Sci. B.

[8] Amaral S., Oliveira P.J., Ramalho-Santos J. (2008). Diabetes and the impairment of reproductive function: Possible role of mitochondria and reactive oxygen species. Curr. Diabetes Rev..

[9] Nna V.U., Abu Bakar A.B., Ahmad A., Eleazu C.O., Mohamed M. (2019). Oxidative stress, NF-κB-mediated inflammation and apoptosis in the testes of streptozotocin-induced diabetic rats: Combined protective effects of malaysian propolis and metformin. Antioxidants.

[10] Qadiri A., Mirzaei B.F., Hamidian G., Zavvari O.Z., Ahmadi M., Oghbaei H., Mehri K., Vatankhah A.M., Keyhanmanesh R. (2019). Administration of troxerutin improves testicular function and structure in type-1 diabetic adult rats by reduction of apoptosis. Avicenna J. Phytomed..

[11] Khosravi Z., Sedaghat R., Baluchnejadmojarad T., Roghani M. (2019). Diosgenin ameliorates testicular damage in streptozotocin-diabetic rats through attenuation of apoptosis, oxidative stress, and inflammation. Int. Immunopharmacol..

[12] Orman D., Vardi N., Ates B., Taslidere E., Elbe H. (2015). Aminoguanidine mitigates apoptosis, testicular seminiferous tubules damage, and oxidative stress in streptozotocin-induced diabetic rats. Tissue Cell.

[13] Zheng Y.C., Feng Y.L., Wang Y.H., Kong L.J., Zhou M.S., Wu M.M., Liu C.Y., Weng H.C., Wang H.W. (2021). Islet transplantation ameliorates diabetes-induced testicular interstitial fibrosis and is associated with inhibition of TGF-β1/Smad2 pathway in a rat model of type 1 diabetes. Mol. Med. Rep..

[14] Shiraishi K., Takihara H., Naito K., Aktuelle U. (2003). Quantitative analysis of testicular interstitial fibrosis after vasectomy in humans. Aktuelle Urol..

[15] Shen N., Li X., Zhou T., Bilal M.U., Du N., Hu Y. (2014). Shensong yangxin capsule prevents diabetic myocardial fibrosis by inhibiting TGF-β1/smad signaling. J. Ethnopharmacol..

[16] Li J., Kang S.W., Kim J.L., Sung H.Y., Kwun I.S., Kang Y.H. (2010). Isoliquiritigenin entails blockade of TGF-β1-smad signaling for retarding high glucose-induced mesangial matrix accumulation. J. Agric. Food Chem..

[17] Gina J.R., Nancy S.W., Andrea R.R., Curtiss B.C. (2003). Chromium as adjunctive treatment for type 2 diabetes. Ann. Pharmacother..

[18] Walter M. (1993). Chromium in human nutrition: A review. J. Nutr..

[19] Amoikon E.K., Fernandez J.M., Southern L.L., Thompson D.L., Ward T.L., Olcott B.M. (1995). Effect of chromium tripicolinate on growth, glucose tolerance, insulin sensitivity, plasma metabolites, and growth hormone in pigs. J. Anim. Sci..

[20] Qi S.S., Zheng H.X., Jiang H., Yuan L.P., Dong L.C. (2020). Protective effects of chromium picolinate against diabetic-induced renal dysfunction and renal fibrosis in streptozotocin-induced diabetic rats. Biomolecules.

[21] Wojciechowska J., Krajewski W., Bolanowski M., Kręcicki T., Zatoński T. (2016). Diabetes and cancer: A review of current knowledge. Exp. Clin. Endocrinol. Diabetes.

[22] Oladipo G.O., Nlekerem C.M., Ibukun E.O., Kolawole A.O. (2018). Quail (*Coturnix japonica*) egg yolk bioactive components attenuate streptozotocin-induced testicular damage and oxidative stress in diabetic rats. Eur. J. Nutr..

[23] La-vignera S., Condorelli R., Vicari E., Dagata R., Calogero A.E. (2012). Diabetes mellitus and sperm parameters. J. Androl..

[24] Zhao Y., Huang W., Wang J., Chen Y., Huang W., Zhu Y. (2018). Taxifolin attenuates diabetic nephropathy in streptozotocin-induced diabetic rats. Am. J. Transl. Res..

[25] Liu Y., Yang Z., Kong D., Zhang Y., Yu W., Zha W. (2019). Metformin ameliorates testicular damage in male mice with streptozotocin-induced type 1 diabetes through the PK2/PKR pathway. Oxid. Med. Cell. Longev..

[26] He W., Liu H., Hu L., Wang Y., Huang L., Liang A., Wang X., Zhang Q., Chen Y., Cao Y. (2021). Icariin improves testicular dysfunction via enhancing proliferation and inhibiting mitochondria-dependent apoptosis pathway in high-fat diet and streptozotocin-induced diabetic rats. Reprod. Biol. Endocrinol..

[27] Hu Y., Chen S., Yan W., Ji L., Shao M., Sun Z., He D., Zhang L., Xia Z., Li X. (2023). Rape bee pollen alleviates renal tissue damage in diabetic rats via anti-inflammation, anti-oxidation, and modulating gut microbiota. eFood.

[28] Koroglu Aydın P., Karabulut-Bulan O., Bugan I., Turkyilmaz I.B., Altun S., Yanardag R. (2022). The protective effect of metformin against testicular damage in diabetes and prostate cancer model. Cell Biochem. Funct..

[29] Maresch C.C., Stute D.C., Alves M.G., Oliveira P.F., Kretser D.M., Linn T. (2018). Diabetes-induced hyperglycemia impairs male reproductive function: A systematic review. Hum. Reprod. Update.

[30] Jiang Z.H., Chen C., Wang J., Xie W., Wang M., Li X., Zhang X. (2016). Purple potato (*Solanum tuberosum* L.) anthocyanins attenuate alcohol-induced hepatic injury by enhancing antioxidant defense. J. Nat. Med..

[31] Qi S., He J., Dong L., Yuan L.P., Wu J., Zu Y.X., Zheng H. (2020). Cyanidin-3-glucoside from black rice prevents renal dysfunction 503 and renal fibrosis in streptozotocin-diabetic rats. J. Funct. Foods.

[32] Shi G.J., Zheng J., Han X.X., Jiang Y.P., Li Z.M., Wu J., Chang Q., Niu Y., Sun T., Li Y.X. (2018). *Lycium barbarum* polysaccharide attenuates diabetic testicular dysfunction via inhibition of the PI3K/Akt pathway-mediated abnormal autophagy in male mice. Cell Tissue Res..

[33] Liu H., Lin S., Lv Q., Yang Q., Wu G., Hu J., Yang J. (2017). Taurine recovers testicular steroidogenesis and spermatogenesis in streptozotocin-induced diabetic rats. Adv. Exp. Med. Biol..

[34] Schoeller E.L., Schon S., Moley K.H. (2012). The effects of type 1 diabetes on the hypothalamic, pituitary and testes axis. Cell Tissue Res..

[35] Cameron D.F., Murray F.T., Drylie D.D. (1985). Interstitial compartment pathology and spermatogenic disruption in testes from impotent diabetic men. Anat. Rec..

[36] Al-Megrin W.A., El-Khadragy M.F., Hussein M.H., Mahgoub S., Abdel-Mohsen D.M., Taha H., Bakkar A.A.A., Abdel-Moneim A.E., Amin H.K. (2020). Green *Coffea arabica* extract ameliorates testicular injury in high-fat diet/streptozotocin-induced diabetes in rats. J. Diabetes Res..

[37] Ahmed H.H., Abd M.D., Abdel A.E., Hadeer A.A. (2017). Pre-clinical study for the antidiabetic potential of selenium nanoparticles. Biol. Trace Elem. Res..

[38] Roy S., Metya S.K., Rahaman N., Sannigrahi S., Ahmed F. (2014). Ferulic acid in the treatment of post-diabetes testicular damage: Relevance to the down regulation of apoptosis correlates with antioxidant status via modulation of TGF-β1, IL-1β and Akt signalling. Cell Biochem. Funct..

[39] Christian H., Alena R., Elizabeth W., Chen J., Schneuder A., Long S.A., Wei S., Rawlings R., Kinsman M., Evanko S.P. (2016). Enhanced T cell responses to IL-6 in type 1 diabetes are associated with early clinical disease and increased IL-6 receptor expression. Sci. Transl. Med..

[40] Maresch C.C., Stute D.C., Ludlow H., Hammers H.P., Kretser D.M., Hedher M.P., Linn T. (2017). Hyperglycemia is associated with reduced testicular function and activin dysregulation in the Ins2 Akita+/− mouse model of type 1 diabetes. Mol. Cell. Endocrinol..

[41] Nasiri K., Akbari A., Nimrouzi M., Ruyvaran M., Mohamadian A. (2021). Safflower seed oil improves steroidogenesis and spermatogenesis in rats with type II diabetes mellitus by modulating the genes expression involved in steroidogenesis, inflammation and oxidative stress. J. Ethnopharmacol..

[42] Atta M.S., Almadaly E.A., El-Far A.H. (2017). Thymoquinone defeats diabetes-induced testicular damage in rats targeting antioxidant, inflammatory and aromatase expression. Int. J. Mol. Sci..

[43] Li Y., Zhan M., Li J., Zhang W., Shang X. (2023). Lycopene alleviates lipopolysaccharide-induced testicular injury in rats by activating the PPAR signaling pathway to integrate lipid metabolism and the inflammatory response. Transl. Androl. Urol..

[44] Kanter M., Aktas C., Erboga M. (2012). Protective effects of quercetin against apoptosis and oxidative stress in streptozotocin-induced diabetic rat testis. Food Chem. Toxicol..

[45] Koh P.O. (2008). Streptozotocin-induced diabetes increases the interaction of Bad/Bcl-XL and decreases the binding of pBad/14-3-3 in rat testis. Life Sci..

[46] Zhao Y.G., Tan Y., Dai J.Y., Li B., Guo L.P., Cui J.W., Wang G.J., Shi X., Zhang X., Mellen N. (2010). Exacerbation of diabetes-induced testicular apoptosis by zinc deficiency is most likely associated with oxidative stress, p38 MAPK activation, and p53 activation in mice. Toxicol. Lett..

[47] AlAmri O.D., Albeltagy R.S., Akabawy M.A., Mahgoub S., Abdel-Mohsen D.M., Abdel-Mohsen A.E., Amin H.K. (2020). Investigation of antioxidant and anti-inflammatory activities as well as the renal protective potential of green coffee extract in high fat-diet/streptozotocin-induced diabetes in male albino rats. J. Funct. Foods.

[48] Cayan S., Dusmez D., Bozlu M., Gorur S., Akbay E. (2001). Human testicular mast cells and fibrosis in infertile men. Fertil. Steril..

[49] Voelker J., Berg P.H., Sheetz M., Duffin K., Shen T., Moser B., Greene T., Blumenthal S.S., Rychlik I., Yagil Y. (2017). Anti-TGF-β1 antibody therapy in patients with diabetic nephropathy. J. Am. Soc. Nephrol..

[50] Salama N., Tsuji M., Tamura M., Kagawa S. (2001). Transforming growth factor (beta1) in testes of aged and diabetic rats: Correlation with testicular function. Arch. Androl..

[51] Kabel A.M. (2018). Zinc/alogliptin combination attenuates testicular toxicity induced by doxorubicin in rats: Role of oxidative stress, apoptosis and TGF-β1/NF-κB signaling. Biomed. Pharmacother..

[52] Marion L., Petra K. (2002). Integration of the TGF-β pathway into the cellular signalling network. Cell. Signal..

[53] Gonzalez C., Matzkin M., Frungieri M., Terradas C., Ponzio R., Puigdomenech E., Levalle O., Calandra R.S., Gonzalez-Calvar S.I. (2010). Expression of the TGF-beta1 system in human testicular pathologies. Reprod. Biol. Endocrinol..

